# The short-term health and psychosocial impacts of domestic energy efficiency investments in low-income areas: a controlled before and after study

**DOI:** 10.1186/s12889-017-4075-4

**Published:** 2017-01-31

**Authors:** Charlotte N. B. Grey, Shiyu Jiang, Christina Nascimento, Sarah E. Rodgers, Rhodri Johnson, Ronan A. Lyons, Wouter Poortinga

**Affiliations:** 1 Welsh School of Architecture, Cardiff University, Bute Building, King Edward VII Avenue, Cardiff, CF10 3NB Wales, UK; 2 School of Social Sciences, Cardiff University, Glamorgan Building, King Edward VII Avenue, Cardiff, CF10 3WT UK; 3 Farr Institute, Swansea University Medical School, Swansea University, Singleton Park, Swansea, SA2 8PP Wales, UK; 4 School of Psychology, Cardiff University, 70 Park Place, Cardiff, CF10 3AT Wales, UK

**Keywords:** Fuel poverty, Energy efficiency, Community-based study, Health, Psychosocial

## Abstract

**Background:**

Research suggests that living in fuel poverty and cold homes contributes to poor physical and mental health, and that interventions targeted at those living in poor quality housing may lead to health improvements. However, little is known about the socio-economic intermediaries and processes that contribute to better health. This study examined the relationship between energy efficiency investments to homes in low-income areas and mental and physical health of residents, as well as a number of psychosocial outcomes likely to be part of the complex relationship between energy efficiency measures and health outcomes.

**Methods:**

A quasi-experimental field study with a controlled pretest-posttest design was conducted (intervention *n* = 364; control *n* = 418) to investigate the short-term health and psychosocial impacts of a domestic energy efficiency programme that took place across Wales between 2013 and 2015. Survey data were collected in the winters before and after installation of energy efficiency measures, including external wall insulation. The study used a multilevel modelling repeated measures approach to analyse the data.

**Results:**

The energy efficiency programme was not associated with improvements in physical and mental health (using the SF-12v2 physical and mental health composite scales) or reductions in self-reported respiratory and asthma symptoms. However, the programme was associated with improved subjective wellbeing (B = 0.38, 95% CI 0.12 to 0.65), as well as improvements in a number of psychosocial outcomes, including increased thermal satisfaction (OR = 3.83, 95% CI 2.40 to 5.90), reduced reports of putting up with feeling cold to save heating costs (OR = 0.49, CI = 0.25 to 0.94), fewer financial difficulties (B = −0.15, 95% CI -0.25 to -0.05), and reduced social isolation (OR = 0.32, 95% CI 0.13 to 0.77).

**Conclusion:**

The study showed that investing in energy efficiency in low-income communities does not lead to self-reported health improvements in the short term. However, investments increased subjective wellbeing and were linked to a number of psychosocial intermediaries that are conducive to better health. It is likely that better living conditions contribute to improvements in health outcomes in the longer term. Better understanding of the impacts on recipients of energy efficiency schemes, could improve targeting of future fuel poverty policies.

## Background

It is established that fuel poverty and living in cold homes can contribute to adverse physical and mental health. Poor respiratory health, asthma, and common mental disorders have been associated with living in damp, cold housing [1, 2]. Evidence suggests that energy efficiency interventions targeted at those at risk of fuel poverty and living in poor quality housing may lead to health improvements. In particular, affordable warmth interventions have the potential to improve general, respiratory and mental health outcomes, and more so when targeted at vulnerable groups [3–5]. The literature suggests that there is evidence that housing investment can lead to health improvements, particularly when thermal conditions inside the home are improved. However, the evidence of impacts on different health conditions are not consistently reported [6]. There is a need therefore for large scale intervention studies to fill the gap in knowledge about the health impacts of housing improvement [6].

Currently there is some evidence, predominantly from smaller, qualitative studies, that suggests that affordable warmth interventions may have psychosocial benefits, that can act as an intermediary indication of the potential for longer term health impacts [4, 7, 8]. Most larger studies have focused on a limited number of health outcomes, and largely ignored psychosocial intermediaries and processes [4], indicating a need for a large scale study to explore the effect of improving thermal conditions inside homes on the wider determinants of health.

The aim of this paper is to examine the associations between an affordable warmth intervention that took place in low income neighbourhoods across Wales, and the impacts on 418 residents living in these properties compared to a similar sized control group. The study investigated the short-term health and psychosocial impacts of domestic energy-efficiency investments in low-income communities; more specifically, to determine their impacts on (1) physical and mental health (using SF12v2), self-reported respiratory and asthma symptoms, and subjective wellbeing, as well as on (2) the psychosocial outcomes of experienced fuel poverty, financial difficulties and stress, food security, housing conditions, and social isolation.

### Relationship between mental and physical health and fuel poverty risk

While there is consistent evidence that poor housing quality is associated with poor physical and mental health of the occupants, much of the current evidence on health effects from poor housing is still predominantly cross-sectional [4]. However, those living in poor housing are also most likely to be socio-economically deprived and suffer long-term chronic ill health. Cross-sectional research is not able to shed light on changes in outcomes resulting from improvements or the direction of the relationship between housing and health [4]. Another factor that may confound results is that vulnerable groups within the population are disproportionately likely to live in poor quality housing, be at risk from low incomes, and spend large amounts of time within the home environment [9].

Improving the energy efficiency of homes in a bid to reduce fuel poverty and improve warmth has been shown to be able to lead to improvements in health. In a systematic review of the literature, Thomson et al. evaluated 19 studies, and found that warmth and energy efficiency interventions can produce improvements in general health, respiratory health, and mental health, with studies targeting those with inadequate warmth and existing chronic respiratory conditions showing the greatest improvements [4].

### Relationship between socioeconomic determinants of health and fuel poverty risk

The health and life chances of low-income households are affected by living in energy inefficient housing at various levels. Fuel poverty may negatively affect health and mental wellbeing *directly* through low indoor temperatures, and more *indirectly* through social problems such as social isolation, restricted use of living space (*spatial shrink*), financial trade-offs (leading to the ‘*heat or eat’* dilemma), and financial stress from a constrained budget and competing expenses, with the potential for debt problems [3].

The processes of living in cold homes or in fuel poverty leading to adverse social outcomes is more complicated to measure, due to the more subjective nature of the experience of psychosocial outcomes [3]. Very few studies have attempted to further explore how these socio-economic processes might link housing quality with physical and mental health [10]. However, it is likely that improved psychosocial outcomes resulting from affordable warmth interventions will have a health promoting effect in the longer term [6], and therefore act as a valuable proxy indication of the potential for health improvements [4].

The literature suggests two interrelated pathways between affordable warmth interventions and improved mental and physical health [7, 11, 12]. The first pathway is where energy efficiency improvements to homes may lead to better *thermal living conditions* through improved indoor air temperature and decreased humidity, both contributing to reduced damp-related housing problems [13]. Warmer, drier homes can contribute to improved respiratory health, and also better mental health through improved thermal satisfaction [14], expanded living space, and reduced social isolation [15]. The second pathway is where energy efficiency measures also contribute to improved wellbeing by making heating more *affordable* [2]. Reduced spending on heating bills alleviates financial stress and fuel poverty among low-income households [8, 16], and helps to free financial resources for better food security [17, 18] and reduced social isolation [19]. According to Liddell and Guiney [12] the two pathways act as cumulative stressors where vulnerability to poor mental health increases when people experience multiple stressors relating to thermal (dis)comfort and fuel poverty.

### Public policy and fuel poverty

Fuel poverty is a social issue that UK and Devolved Governments aim to eradicate through a range of policy initiatives. These initiatives rely on correctly identifying those living in fuel poverty in order to be able to target energy efficiency improvements to those in greatest need. However, any measurement of fuel poverty based on income assessment is intrusive, and so policies often rely on proxy measures of fuel poverty in order to decide where to target their programmes, but that causes difficulties in that it is likely that this will lead to incorrect identification of a proportion of the fuel poor [3]. This would lead to households not in fuel poverty receiving energy efficiency measures through policy-led programmes, and other households living in fuel poverty missing out.

It is also important to consider that associations between improving the energy efficiency of homes and the health of occupants may not be simple relationships of cause and effect. Fuel poverty is intrinsically linked to wider issues of deprivation, including chronic physical and mental ill health, financial stress, and socio-economic problems. This makes it difficult to uncover the positive impacts resulting from the delivery of an affordable warmth scheme, but also assessing how to deliver the scheme to the recipients who would benefit the most. It is therefore essential to understand the processes underlying the links between energy efficiency investments and different mental and physical health outcomes. This allows a better understanding of the wider impacts of fuel poverty programmes, but also improved knowledge on how future policies can be improved. The work reported here contributes to the evidence that fuel poverty interventions make a contribution to the wider determinants of health, and suggests that it is likely that this will lead to health improvements over the long term through these socioeconomic intermediary pathways and also suggest that these intermediaries could be used to better target recipients of fuel poverty policies.

## Methods

### Study design

The quasi-experimental controlled pretest-posttest study took place between 2013 and 2015, as part of an evaluation of a Welsh Government-led energy-efficiency investment programme (Arbed). The communities eligible for the programme were selected by policymakers on the basis of proxies of fuel poverty, including area deprivation, mixed tenure, and a high proportion of hard-to-heat, hard-to-treat homes. Matched control areas were identified with help of Local Authorities, using the same selection criteria. In total, 24 intervention and 23 control areas were included in this research. The departmental research ethics committee at Cardiff University gave approval for the study.

### Sampling and recruitment

The study focused on energy-efficiency schemes that were delivered in 2014 and 2015. Any adult resident living in the selected intervention and control areas was eligible for inclusion and a purposive sampling strategy was used to recruit participants. The assignment of participants to the intervention and control groups was not randomised because the researchers had no control over inclusion to the programme. Quantitative data were collected through self-completion questionnaires administered via a drop-off-and-collect method [20].

Baseline (pre-intervention) data were collected during the winters of 2013–14 (November 2013 to March 2014) and 2014–2015 (November 2014 to January 2015) before any energy-efficiency work had started. Data collection was coordinated with the scheduled delivery of the different schemes. Follow up (post-intervention) data were collected during the winters of 2014–15 (November 2014 and January 2015) and 2015–2016 (November and December 2015) after all work was completed. The improvement work for the different intervention schemes were conducted throughout the 2 years. The follow up data were therefore collected between 1 and 10 months post intervention. Records of the completed properties were provided by the two scheme managers. Data for the intervention and matched control areas were collected during the same time periods. Data for one scheme could not be collected in two subsequent winters due to delays in the delivery of the energy-efficiency improvements. In this case, the follow up data for both the intervention and its matched control area were collected with a 2 year gap, but in the winter immediately following the completion of work.

Questionnaires were distributed to eligible households across the intervention and control areas. In total, 1,508 participants were recruited in the pre-intervention period (656 from the intervention and 852 from the control areas). Of the 1,508 baseline participants, 220 did not consent to be recontacted, and a further 506 participants were lost to follow up, reflecting an overall attrition rate of 48.1%. A loss to follow up analysis showed that there were a number of socio-economic differences between the respondents included in the final study sample and those who dropped out in between baseline and follow up. In terms of health outcomes, the respondents lost to follow-up were more likely to be at risk of common mental disorders, as measured by the MCS of the SF12v2. However, there were no differences with regard to physical health, as measured by the PCS of the SF12v2, subjective wellbeing, and self-reported respiratory and asthma symptoms.

Not every eligible household within the scheme areas elected to have energy-efficiency work done to their house. Respondents from intervention areas who did not have energy efficiency work done to their home became part of the control group (*n* = 81) making the total intervention *n* = 364 and controls *n* = 418. The final sample numbers provided 80% statistical power to detect effect sizes of d = 0.18 at the 5% significance level, in line with effect sizes observed in comparable field studies examining the health effects of housing improvements [4, 21, 22].

### Intervention

The intervention programme aimed to improve the energy performance of hard-to-heat, hard-to-treat homes in low-income areas across Wales, as part of its national fuel poverty reduction policies. Energy efficiency measures included external wall insulation, central heating system upgrades (boilers and radiators), and the connection of off-gas communities to the mains gas network. The intervention was Government-led and managed by two scheme managers, who determined the most appropriate and cost-effective measures at a scheme-by-scheme basis. All measures were free to householders who elected to receive them.

### Measures

Self-reported health outcomes were: physical and mental health status as measured by the SF12v2 Physical (PCS) and Mental (MCS) Health Composite Scales, respiratory and asthma symptoms, and subjective wellbeing. The SF-12v2 is a validated questionnaire for measuring health-related quality of life [23]. Respiratory symptoms were measured using items adapted from Fisk et al. [24] and the World Health Organization [25], asthma symptoms with the short version of the European Community Respiratory Health Survey [26], and subjective well-being with four questions developed by the Office for National Statistics [27].

The psychosocial outcomes were measured as follows. Asking participants whether within in the past 12 months they had put up with feeling cold to save heating costs was used to indicate fuel poverty [28]. The financial difficulties questions measured how often respondents had difficulties meeting the cost of the four house-related expenses of rent or mortgage payments, repairs or maintenance of home, fuel bills, and credit payments [29]. The four responses were combined into a single scale. Financial stress was measured using a question derived from the INTERHEART study [30]. Food security, conceptualised as economic access to food in terms of quantity, quality, and variety, was measured with three questions from the US Adult Food Security Survey [31]. Thermal satisfaction was measured by asking respondents how satisfied they are with the temperature in the home on a typical winter day. Respondents could answer using a 5-point scale. Housing conditions were assessed by asking respondents about satisfaction with the current state of repair of their home, and then whether they experienced any of six housing-related problems, such as damp, mould and condensation. Social isolation was measured by asking respondents whether they had been reluctant to invite friends or family home in the last year because of difficulties keeping it warm [32].

### Analysis

A multilevel modelling, repeated measures approach was used to examine the impacts of the energy-performance investments on the different health and psychosocial outcomes. Analyses were conducted with MLwiN version 2.36 [33, 34]. The dataset included two measurement occasions (level 1) nested within individuals (level 2), with the intervention group (intervention versus control) as an individual level factor and measurement occasion (follow up versus baseline) as a within person factor. Only the interaction effects indicating the differential changes between the intervention and control groups are reported. All analyses were conducted with and without adjusting for the a priori selected covariates of gender, age, housing benefit, household income, and smoking status. Cohen’s d was calculated to indicate the size of the interaction effects. The types of models constructed (linear, ordered multinomial, or logistic) depended on the outcome variable. Parameters in all models were estimated using Monte Carlo Markov chains with 50,000 iterations.

## Results

### Sample characteristics

Table 1 summarises the socio-demographic and building characteristics of the final study sample. The intervention and control populations were comparable in terms of socio-demographic and building characteristics, with chi-squared tests showing no significant differences between the two groups for any of the variables listed in Table 1. Because the intervention and control group were largely similar there was a low likelihood of confounding.

**Table 1:** Socio-demographic and building characteristics of the intervention and control groups of the study cohort

Characteristics	Category	Intervention % (*n*)	Control % (*n*)
Socio-demographic characteristics			
Gender	Male	41.2 (145/352)	40.3 (166/412)
	Female	58.8 (207/352)	59.7 (246/412)
Age (years)	Under 25	1.4 (5/362)	2.1 (9/414)
	26–35	6.9 (25/362)	6.0 (25/414)
	36–45	10.8 (39/362)	9.4 (39/414)
	46–54	14.1 (51/362)	11.1 (47/414)
	55–64	30.1 (109/362)	25.1 (104/414)
	65 or above	36.7 (133/362)	45.9 (190/414)
Household composition	Households with no children	79.1 (284/359)	83.1 (339/415)
	Households with children	20.9 (75/359)	16.9 (76/415)
Marital status	Single	13.8 (50/363)	11.3 (47/412)
	Married/cohabiting	55.1 (200/363)	51.6 (214/412)
	Separated/divorced	16.0 (58/363)	21.7 (90/412)
	Widowed	14.9 (54/363)	15.2 (63/412)
Household income	£0-4,999	3.5 (12/339)	3.6 (14/392)
	£5,000-9,999	23.5 (80/339)	25.8 (101/392)
	£10,000-19,999	33.9 (115/339)	35.9 (141/392)
	£20,000-29,999	18.0 (61/339)	15.3 (60/392)
	£30,000 or higher	20.6 (70/339)	19.4 (76/392)
Housing benefits	Yes	25.1 (89/354)	24.4 (98/402)
	No	7.9 (265/354)	75.6 (304/402)
Tenure	Owner occupied	77.5 (276/356)	74.0 (307/415)
	Private rental	3.9 (14/356)	6.7 (28/415)
	Local authority rental	13.8 (49/356)	13.3 (55/415)
	Housing association rental	3.4 (12/356)	5.3 (22/415)
Time lived at current address	Less than one year	3.3 (12/360)	4.3 (18/416)
	1–4 years	9.4 (34/360)	11.8 (49/416)
	5–9 years	14.4 (52/360)	12.7 (53/416)
	More than 9 years	72.8 (262/360)	71.2 (296/416)
Building characteristics			
Building type	Detached house	12.5 (45/360)	12.3 (51/416)
	Semi-detached house	38.1 (137/360)	28.8 (120/416)
	Terraced house	41.1 (148/360)	46.9 (195/416)
	Bungalow	5.0 (18/360)	5.3 (22/416)
	Flat	1.4 (5/360)	6.0 (25/416)
Building age	Before 1919	44.1 (152/345)	40.7 (166/405)
	1919–1945	26.1 (90/345)	24.3 (99/405)
	1945–1965	17.4 (60/345)	23.5 (96/405)
	1965–1979	7.5 (26/345)	6.1 (25/405)
	1980 or later	4.9 (17/345)	5.4 (22/405)
Number of bedrooms	One	1.7 (6/358)	5.4 (22/409)
	Two	19.8 (71/358)	20.8 (85/409)
	Three	68.2 (244/358)	65.3 (267/409)
	Four or more	10.4 (37/358)	8.5 (35/409)
Arbed energy efficiency measures	External wall insulation	71.7 (261/364)	---
	Full central heating	39.0 (138/354)	---
	Voltage Optimiser	44.4 (159/358)	---
	Heating control	28.6 (101/353)	---
	Connection to mains gas	13.9 (49/353)	---

Note: Denominators vary due to missing data

### The impacts of the intervention on health outcomes

The study found no evidence that the intervention had a significant impact on physical and mental health (Table 2). Although there was a small increase in PCS scores for the intervention group, the change was not significantly different from the small reduction observed for the control group. There was a slight increase in the MCS scores for the intervention group. However, the increase for the intervention group was similar to the increase observed for the control group. A small reduction in the number of reported respiratory symptoms between baseline and follow up for the intervention group could be seen. However, the change did not statistically differ from the one observed for the control group. Similarly, the reduction in the number of reported asthma symptoms for the intervention group did not significantly differ from the control group.

**Table 2:** Health outcomes at baseline and follow-up for the intervention and control group

		Intervention		Control		Effect size		Unadjusted			Adjusted^(a)^		
Outcome	Scale	Baseline M (SD)	Follow up M (SD)	Baseline M (SD)	Follow up M (SD)	Cohen’s d^(c)^	Model^(b)^	B	SE	p	B	SE	p
MCS (mental health)	0–100	44.81 (12.56)	45.62 (11.94)	46.02 (12.06)	46.85 (12.36)	0.005	L	−0.059	0.789	0.940	0.003	0.812	1.000
PCS (physical health)	0–100	42.90 (13.94)	43.11 (13.80)	42.25 (14.47)	41.48 (14.24)	0.107	L	0.987	0.664	0.137	0.976	0.669	0.145
Respiratory symptoms	0–11	2.97 (2.74)	2.91 (2.74)	2.94 (2.67)	3.05 (2.77)	0.061	L	−0.155	0.192	0.419	−0.141	0.202	0.485
Asthma symptoms	0–9	2.34 (2.55)	2.20 (2.45)	2.23 (2.50)	2.24 (2.51)	0.051	L	−0.088	0.247	0.722	−0.133	0.253	0.600
Subjective wellbeing	0–10	6.55 (2.50)	6.89 (2.25)	6.96 (2.42)	6.92 (2.42)	0.200	L	0.375	0.134	0.005	0.384	0.134	0.004

Note: ^(a)^adjusted for gender, age, housing benefit, income, and smoking status; ^(b)^L = linear, O = ordinal, B = binomial; ^(c)^Cohen’s d effect sizes: small 0.20, medium 0.50, and large 0.80

Table 2 shows an increase in subjective wellbeing for the intervention group, but a slight decrease for the control group. This represents a significant interaction, which remained after controlling for the covariates (B = 0.38, 95% CI 0.12 to 0.65). This suggests that the intervention had a positive impact on overall feelings of wellbeing.

### The impacts of the intervention on the psychosocial outcomes

Analyses indicate that the intervention had an impact on a number of psychosocial outcomes, including financial difficulties, thermal satisfaction, satisfaction with state of home repair, the number of housing problems, and social isolation (Table 3). The number of respondents reporting putting up with feeling cold to save heating costs decreased for the intervention group. A smaller decrease was observed for the control group. The effect remained significant after controlling for the covariates (OR = 0.49, CI = 0.25 to 0.94). The effect size of the (unadjusted) interaction was small (Cohen’s d = 0.15). Self-reported financial difficulties decreased to a greater extent in the intervention group than in the control group. The effect remained after controlling for the covariates (B = -0.15, 95% CI −0.25 to −0.05), with a small effect size (Cohen’s d = 0.20). A significant interaction effect was observed for thermal satisfaction, which remained after controlling for the covariates (OR = 3.83 95% CI 2.40 to 5.90), with a medium effect size (Cohen’s d = 0.46). Although both the intervention and control groups saw an increase in thermal satisfaction, the increase was greater for the intervention group. Significant interaction effects were observed for the two housing conditions variables. Both effects remained significant after adjusting for the covariates. Satisfaction with the state of repair of their home increased for the intervention group, but decreased for the control group (OR = 3.87 95% CI 2.51 to 5.96). The number of reported housing problems decreased for both the intervention and control groups, but decreased for a greater extent in the intervention group (OR = 0.33, 95% CI 0.21 to 0.52). The effects were small-to-medium sized (Cohen’s d = 0.44 and 0.39, respectively).

**Table 3:** Psychosocial outcomes at baseline and follow-up for the intervention and control group

		Intervention		Control		Effect size		Unadjusted			Adjusted^(a)^		
Outcome	Scale	Baseline M (SD)	Follow up M (SD)	Baseline M (SD)	Follow up M (SD)	Cohen’s d^(c)^	Model^(b)^	B	SE	p	B	SE	p
Fuel poverty													
Putting up with feeling cold to save heating costs	0–1	0.63 (0.48)	0.45 (0.50)	0.57 (0.50)	0.46 (0.50)	0.153	B	−0.720	0.324	0.026	−0.717	0.334	0.032
Financial difficulties	1–4	1.93 (0.90)	1.67 (0.73)	1.74 (0.75)	1.65 (0.73)	0.204	L	−0.148	0.047	0.002	−0.149	0.050	0.003
Financial stress	1–5	2.96 (1.40)	2.60 (1.35)	2.81 (1.38)	2.58 (1.36)	0.108	O	−0.381	0.213	0.074	−0.403	0.220	0.067
Food security	1–4	3.51 (0.77)	3.61 (0.73)	3.55 (0.77)	3.59 (0.73)	0.117	L	0.057	0.035	0.093	0.063	0.036	0.080
Thermal satisfaction	1–5	3.26 (1.28)	4.04 (1.06)	3.60 (1.26)	3.82 (1.20)	0.462	O	1.319	0.216	0.000	1.342	0.219	0.000
Housing conditions													
Satisfaction with state of home repair	1–5	3.45 (1.15)	3.85 (1.10)	3.71 (1.16)	3.63 (1.22)	0.440	O	1.342	0.222	0.000	1.352	0.221	0.000
Number of housing problems	0–6	1.88 (1.53)	1.20 (1.32)	1.49 (1.53)	1.32 (1.45)	−0.386	O	−1.065	0.221	0.000	−1.097	0.225	0.000
Social Isolation	0–1	0.24 (0.43)	0.12 (0.33)	0.18 (0.39)	0.14 (0.34)	−0.195	B	−1.052	0.429	0.014	−1.149	0.456	0.012

Note: ^(a)^adjusted for gender, age, housing benefit, income, and smoking status

^(b)^L = linear, O = ordinal, B = binomial

^(c)^Cohen’s d effect sizes: small 0.20, medium 0.50, and large 0.80

Finally, a small significant interaction effect was found for social isolation (Cohen’s d = 0.19), where the greatest improvement in feeling reluctant to invite friend or family to their home because of difficulties keeping it warm, was seen in the intervention group. This effect remained after controlling for the covariates (OR = 0.32, 95% CI = 0.13 to 0.77).

## Discussion

We present the results of one of the first large controlled before and after quasi-experimental studies that investigates both the short-term health and psychosocial impacts of energy-efficiency investments. The study did not find evidence that investments in energy efficiency improve respiratory, mental or physical health in the short term. However, those who received energy-efficiency measures reported improved subjective wellbeing compared to the control group, as well as improvements in a number of psychosocial outcomes that are indicative of a positive impact to wider determinants of health. Respondents who received the intervention reported fewer financial difficulties, higher thermal satisfaction, and higher satisfaction with the improvement of their homes. They were also less reluctant to invite friends or family to their homes after the improvements, reducing their social isolation.

Our results show the importance of considering socioeconomic factors in addition to health outcomes when evaluating affordable warmth interventions. The observed changes in the psychosocial outcomes are indicative of improved living conditions and quality of life. We would expect that these improvements in these wider determinants of health would contribute to better physical and mental health in the longer term.

Previous studies have shown that improvements in general health, respiratory, and mental health are possible from improving the energy efficiency of fuel poor homes [4]. However, not all studies have shown the same health impacts, and in some cases no or negative effects were observed [4]. These contrasting results may be due to a number of factors, including the follow up time period since the intervention [4], age and health status of the residents at baseline [22], and type and extent of intervention measures delivered. In a recent meta-analysis, Maidment and colleagues [35] showed that the impact of energy efficiency interventions on health is dependent on a number of factors, including the measurement of health, the type of study participant and setting, as well as the scale and type of the intervention. There may also be latent mechanisms at play, and contextual issues which may contribute to differences in outcomes [36]. An example is an intervention study that found improvements in respiratory health, but the measure installed was a non-polluting heating system that alleviated asthma symptoms in children that had the dual effect of raising temperature but also lowering asthma-triggering pollutants [37]. The systematic review by Thomson and colleagues reported that a limitation of most studies investigating long term physical and mental health impacts of housing improvements were their limited follow up periods [4, 38]. This study did not find any significant impact on health which is comparable to other short-term studies [22]. Thomson and colleagues concluded that investments to improve warmth in homes can lead to health improvements, particularly if specifically targeted towards recipients with chronic respiratory health, however, impacts were less clear in programmes which were area rather than individual-needs based [4]. Another complexity in assessing health outcomes is that the energy efficiency interventions received by households not only vary between different studies, but also households within the study [4], which is a limitation of any quasi-experimental study.

Research examining the impacts of energy efficiency investments on wider determinants of health associated with energy efficiency improvements to homes have so far predominantly been qualitative. These studies have suggested that, similar to our findings, a number of socio-economic factors have been improved as a result of affordable warmth interventions, and that they are predominantly related to *affordability* and *improved (thermal) living conditions.*

We found that the intervention had an impact on a number of psychosocial outcomes, including putting up with feeling cold to save heating costs, financial difficulties, thermal satisfaction, satisfaction with state of home repair, the number of housing problems, and social isolation. Our results support the findings of previous qualitative studies, and provide quantitative evidence of the potential mechanisms to improve health in the long term supporting the need for fuel poverty programmes.

A reduction in fuel bills has been shown in several studies after energy efficiency measures have been installed [39–41], the resulting benefits include increased affordability which has been shown reduce financial stress and anxiety [42–44] and improve disposable income allowing for more flexible expenditure [16]. Making the heating more controllable and efficient, combined with reducing expenditure on fuel, allows residents to heat the home more adequately and evenly [41, 45] improves thermal comfort, and reduce housing problems such as the presence of damp and mould [40, 42, 45, 46]. Improvements in social functioning have been highlighted in several studies [8]. Social isolation may be reduced as the reluctance to invite people over may be reduced following improving thermal conditions in the home [46], and from more available finances which may allow for more social events outside of the home [41].

### Strengths and weaknesses

A distinctive strength of the study is that it included a wide range of psychosocial outcomes alongside a number of health outcome. As such, it built upon the Warm Front health impact evaluation [11], which showed that energy efficiency measures were directly and indirectly associated to better mental health via increased thermal comfort, as well as reduced stress and fuel poverty. In our research, we broadened the evidence base by examining the health and psychosocial impacts through a controlled intervention study. The study was quasi-experimental, as it was not possible to randomise the intervention. The intervention and control groups were however largely comparable, suggesting a low chance of confounding. The size of the study allowed small effects to be detected. A strength of the study is the depth of data collected.

Researchers evaluating complex social interventions are faced with several challenges [47, 48]. The study involved an evaluation of an external, policy-led programme, and the researchers did not have control over the content or delivery of the programme. A few issues arise from this. Firstly, not all eligible households elected to have energy-efficiency work done to their house, and some respondents may have independently chosen to undertake energy-efficiency improvements to their home outside the programme, therefore diluting the effects of the intervention [8, 10] There is, however, no evidence that this happened at a large scale in the study.

Secondly, interventions targeting at-risk populations provided clearer health benefits than area-based programmes [4, 22]. While the programme was area-based, it did focus on communities at risk of fuel poverty, in areas of high levels of deprivation and poor quality housing. These households are more likely to benefit from the intervention in terms of health, however, as Curl and colleagues discuss, living in low income, deprived communities means that individuals are exposed to a multitude of factors contributing to ill health over a lifetime, meaning a short term effect from housing improvements is difficult to identify from other factors contributing to, or improving, health inequalities [49].

Thirdly, outcome measures were collected in the winters directly before and after the intervention, meaning that only claims can be made about short-term effects. While most of the improvement work was conducted throughout the 2 years in which the study was conducted, there were a few instances where the data were collected a short as 1 month after the intervention. It is likely that the health impacts will not have been materialised by then. Studies with longer follow up periods are needed to establish the long-term impacts of energy-efficiency investment [4]. However, the path from intervention to changes in health may take many years, and during that time new factors are constantly introduced that may affect the outcome of interest, making it difficult to attribute possible effects to the intervention under investigation [48].

Given that the households receiving the intervention were at higher risk due to poor housing, it would be expected that over time a greater effect would be observed. In order to investigate this, anonymised data linkage of routinely collected health data is being analysed for recipients of the efficiency investment programme that took place between 2010 and 2012 [50, 51]. These results will be reported elsewhere.

## Conclusions

This study examined the associations between housing warmth improvements, health, and psychosocial factors, after controlling for personal characteristics (gender, age, housing benefit, household income, and smoking status). It found that the energy efficiency intervention was significantly associated with improved subjective wellbeing, reduced financial difficulties and social isolation, and increased thermal satisfaction and satisfaction with the home in terms of repair and damp-related housing problems, and that recipients were less likely to have to put up with feeling cold because of the expense associated with heating a home adequately. However, the improvements in energy efficiency did not lead to measurable self-reported physical or mental health improvements during the period of the study. Our results indicate that the policy-led intervention had a positive impact on a number of wider determinants of health that are likely to be part of the pathways between energy efficiency improvements and mental and physical health outcomes, and given the improvements in the psychosocial intermediaries that better health outcomes may materialise in the longer term. It shows that, when designing energy efficiency and/or fuel poverty interventions, it is important to consider these wider social determinants. While energy efficiency investments may not provide immediate health benefits, they do deliver a better quality living environment that is conducive to better health and wellbeing. That is a positive outcome in itself. Targeted demand-led schemes may lead to the biggest health improvements [4], but do not necessary reduce the existing health and social inequalities, because they focus on a relatively small proportion of the population. The principle of *proportionate universalism* may therefore be useful for energy efficiency investments [52]. Actions have the potential to improve the quality of life of the whole population, both in terms of health and the environment, but should be delivered with an intensity that is proportionate to the level of need. The combination of demand-led and more general area-based schemes, as is currently done by the Welsh Government Warm Homes Nest and Arbed programmes, may help ensure that benefits are distributed across society. Finally, future research should concentrate on longer-term impacts, and attempt to distinguish the effects of housing energy improvements from other social, environmental and behavioural factors that contribute to health inequalities and ill health, making use of a wider variety of methods, including data linkage from objectively recorded routine data, or qualitative study.
